# Experimental Study on Rheological, Mechanical Properties and Microstructure of Ultra-High Performance Concrete (UHPC) Mixed with Steel Slag Powder

**DOI:** 10.3390/ma19071463

**Published:** 2026-04-05

**Authors:** Lei Liu, Hao Chen, Xinhua Cai, Jinyang Cui, Wei Guo

**Affiliations:** 1 Department of Hydraulic Engineering, Shaanxi A&F Technology University, Xianyang 712100, China; liulei2018@whu.edu.cn; 2 Zhuhai Chunhe New Materials Research Institute Co., Ltd., Zhuhai 519000, China; 3 College of Civil Engineering, Xijing University, Xi’an 710123, China; 4 State Key Laboratory of Water Resources Engineering and Management, Wuhan University, Wuhan 430072, China; 5 Hydraulic Concrete Institute, China Three Gorges Corporation, Beijing 100038, China

**Keywords:** ultra-high performance concrete, steel slag powder, rheology, mechanical properties, microstructure

## Abstract

In order to promote the high-quality utilization of solid waste—steel slag—this study prepared ground steel slag powder with specific surface areas of 400 m^2^/kg, 500 m^2^/kg and 600 m^2^/kg respectively. Different fineness levels of steel slag powder were used to replace cement to prepare ultra-high performance concrete (UHPC), with replacement rates of 20%, 30% and 40% respectively. The effects of fineness and dosage of steel slag powder on the workability, mechanical properties and microstructure of UHPC were further investigated. The results show that the incorporation of steel slag powder can significantly reduce the yield stress and plastic viscosity of UHPC, thereby increasing its fluidity, but also decreasing its thixotropy. The tensile properties of UHPC mixed with steel slag powder were all superior to those of the reference group. The compressive strength of UHPC prepared by using steel slag powder with a specific surface area of 400 m^2^/kg or 600 m^2^/kg instead of 20% cement was higher than that of the reference group. The compressive strength of UHPC mixed with 600 m^2^/kg specific surface area steel slag powder was generally stronger at the same dosage. At the same fineness, the mechanical properties of UHPC decreased gradually with the increase in steel slag powder content. The recommended dosage for the steel slag powder with a specific surface area of 400 m^2^/kg is 20%, which results in the best comprehensive properties in UHPC. At this time, compared with the reference group, the compressive strength increased by 3.35%, and the tensile strength increased by 20.73%. Moreover, adequate fineness of the steel slag powder can be achieved without excessive grinding energy, which contributes to sustainability.

## 1. Introduction

As a by-product of iron and steel production, steel slag accounts for about 10–15% of crude steel production [1,2]. The chemical composition of steel slag is similar to that of portland cement clinker, mainly including CaO, SiO_2_, Fe_2_O_3_, MgO, and a small amount of Al_2_O_3_, MnO_2_, P_2_O_5_, etc. The mineral composition of steel slag mainly includes C_3_S, *β*-C_2_S, C_4_AF, C_2_F, RO phase (CaO-FeO-MnO-MgO solid solution) and a small amount of *f*-CaO, etc. [3]. The mineral composition of steel slag varies among different steel mills due to differences in raw materials and production processes. However, at present, the extremely low utilization rate of steel slag has caused an increasingly serious negative impact on the environment.

Ultra-high performance concrete (UHPC) is a typical representative of the advanced generation of cement-based materials, characterized by ultra-high strength, high toughness and excellent durability. Since Bache invented UHPC in 1978 [4], it has become the research and application hotspot of many experts, scholars and engineers all over the world because of its excellent mechanical and durability properties [5,6]. UHPC is more and more used in practical projects, such as UHPC bridge decks, curtain walls, and UHPC maintenance and reinforcement of existing reinforced concrete bridges [7]. Using steel slag powder as a mineral admixture to prepare cement-based materials can improve the high-performance utilization of industrial waste-steel slag, reduce environmental damage, and reduce the cost of cement-based materials to some extent [8,9].

Many scholars use steel slag as a mineral admixture instead of cement to prepare normal concrete and study its workability and mechanical properties. Han et al. [10] found that adding steel slag powder to concrete has a negative impact on its performance after five years, such as low compressive strength and high porosity. However, the long-term performance of steel slag powder concrete can be improved, especially at a low water–binder ratio. Pan et al. [11] studied the influence of steel slag powder on the properties of recycled aggregate self-compacting concrete (SCRAC) and found that the early strength growth of SCRAC mixed with steel slag powder was relatively slow. When steel slag powder replaces 10% of cement, the strength of SCRAC is higher than that of the reference group, but when the replacement rate of steel slag powder exceeds 20%, the strength of SCRAC is adversely affected. Zang et al. [12] found that smaller steel slag powder (0–15 μm and 15–30 μm) has a negative impact on the workability of concrete, while relatively coarse steel slag powder particles (30–45 μm and 45–80 μm) have a positive impact. However, when the substitution rate exceeds 20%, 45–80 μm steel slag powder also reduces the workability of concrete. Steel slag powder with a replacement rate of no more than 10% and particle sizeS of 0–15 μm and 45–80 μm is conducive to the development of 7–28 d compressive strength of concrete. The concrete containing 5% steel slag powder of 45–80 μm has the highest compressive strength and lower porosity, with a finer pore structure. Ming et al. [13] found that the physical filling and secondary hydration effects of steel slag powder and zeolite powder made the internal structure of concrete denser and thus had good later strength. Han et al. [14] showed that adding steel slag powder can improve the workability of concrete, reduce plastic viscosity and temperature rise during hardening, and also reduce the compressive strength of concrete. Fang et al. [15] pointed out that when steel slag powder and fly ash replace 25% and 15% of cement respectively, the 7 d and 28 d compressive strength of concrete increased significantly. Zhuo et al. [16] studied the fracture behavior of steel slag powder concrete and showed that adding less than 10% steel slag powder can improve the fracture performance of concrete, which may be because steel slag powder strengthens the bonding performance between aggregate and matrix to a certain extent. Martins et al. [17] found that when steel slag powder replaced 25% of the cement, the mortar had better compressive strength, but the compressive strength decreased linearly with the increase in steel slag powder content. Steel slag powder and other solid waste grinding powders also have an influence on the performance of recycled aggregate concrete. Zhan et al. [18] found that the recycled aggregate concrete containing 20% steel slag powder and 10% waste glass powder had the highest compressive strength and the densest microstructure. The compressive strength at 28 days and 120 days was 28.3% and 22.8% higher than that of the control group, respectively. Tian et al. [19] found that the fluidity of cement paste was the greatest when the content of steel slag powder was less than 20% and 1.3% superplasticizer was added. Wang et al. [20] studied the early shrinkage and mechanical properties of concrete with different cementitious materials, and found that the concrete prepared by replacing 30% cement with steel slag powder, slag and fly ash (mass ratio of 1:2:1) has superior mechanical properties and small early shrinkage. With the increase in steel slag powder content, the performance of concrete decreases. By studying the hydration effect and mechanism of steel slag powder and granulated blast furnace slag powder, Zhao et al. [21] found that steel slag powder and slag powder have a hydration superposition effect, and the strength of the paste is significantly higher than that of the two used alone. When the mass ratio of steel slag powder to slag powder is 2:3 and 1:1, the mechanical properties of the corresponding mortar at 7 d (16.5 MPa) and 28 d (31 MPa) are the best, respectively.

Liu et al. [22] studied the application of steel slag powder and steel slag aggregate in ultra-high performance concrete, and found that UHPC containing steel slag powder had better compressive strength when the cement substitution rate was less than 10%. Steel slag powder as a mineral admixture can improve the fluidity and workability of the paste. Steel slag powder has more continuous reaction activity than cement in the later hydration stage. The cumulative pore volume of steel slag powder UHPC increased significantly with the increase in cement replacement rate. Zhang et al. [8] found that a high steel slag powder content reduces the compressive strength of UHPC, and the negative impact at early age is more obvious, which is consistent with hydration behavior. When 30% of the cement is replaced by steel slag powder, the harmful porosity greater than 100 nm increases from 0.93% to 2.38%, which leads to a decrease in compressive strength. Peng et al. [23] studied the durability and microstructure of UHPC with a high content of steel slag powder and ultra-fine fly ash. The results show that UHPC has high bulk density, low porosity, small pore size and very dense microstructure. Fan et al. [24] studied ultra-high performance concrete with environmental protection steel slag powder, and found that adding a proper amount of steel slag powder (200 kg/m^3^) did not affect the compressive strength of UHPC, and at the same time, it had high workability and low porosity. However, Li et al. [25] studied the influence of steel slag powder and expansive agent on the performance of UHPC, and found that with the increase in steel slag powder content, the workability of UHPC decreases. The suitable contents of steel slag powder and expansive agent are 15% and 5% respectively. The proper combination of steel slag powder and expansive agent can effectively reduce the total shrinkage of UHPC without significantly reducing its workability and mechanical properties.

At present, there are relatively few studies on UHPC mixed with steel slag powder. In this study, steel slag powder with different fineness (specific surface area of 400 m^2^/kg, 500 m^2^/kg and 600 m^2^/kg, respectively) is used to prepare UHPC, and the cement substitution rates are 20%, 30% and 40%, respectively. The workability performance of UHPC mixed with steel slag powder is characterized by testing rheological parameters, and the relationship between fluidity and rheological parameters is analyzed. By testing its mechanical properties and combining them with the microstructure formation mechanism, the internal influence of steel slag powder UHPC microstructure on the mechanical properties is investigated.

## 2. Materials and Methods

### 2.1. Materials

Cement (CEM) adopts P·O 42.5 ordinary portland cement of Rizhao Zhonglian Port Cement Co., Ltd., Rizhao, China. According to the standard GB/T 8074-2008 [26], using the FBT-9 model apparatus for blaine method by Shanghai Yichang Company (Shanghai, China), the specific surface area was measured to be 332 m^2^/kg. Using a Le Chatelier flask, the cement was replaced with an equal volume of anhydrous kerosene, and the density was measured as 3.02 g/cm^3^. Silica fume (SF) is Elkem Microsilica^Ⓡ^940 from Norwegian Ekeng Company’s agent, Zhengzhou, China. The content of SiO_2_ is more than 95%. Fly ash microspheres (FAM) are from Tianjin Zhucheng New Materials Technology Co., Ltd., Tianjin, China. Steel slag is produced in Wulian County, Rizhao, China. It is ground into three kinds of steel slag powder (SSP) with different fineness by the DQM-20L planetary ball mill manufactured by JinTu Electronic Technology Co., Ltd., Xiamen, China. The specific surface areas of steel slag powder 1 (SSP 1), steel slag powder 2 (SSP 2) and steel slag powder 3 (SSP 3) are 400 m^2^/kg, 500 m^2^/kg and 600 m^2^/kg respectively. The main chemical compositions of SSP is shown in Table 1. Except for the SSP, the other raw materials are the same as those mentioned in Reference [27]. 

**Figure 1:**
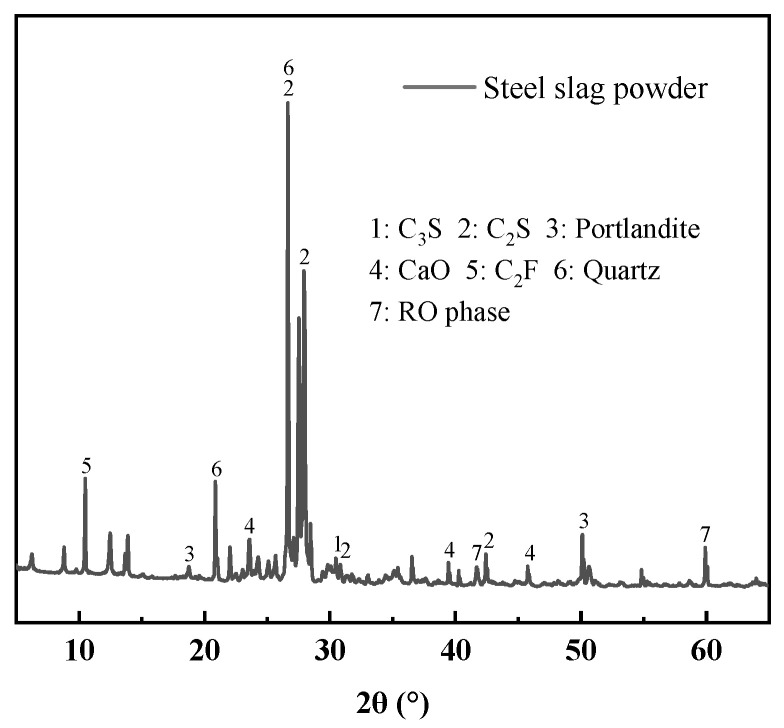
XRD pattern of the steel slag powder.

**Figure 2:**
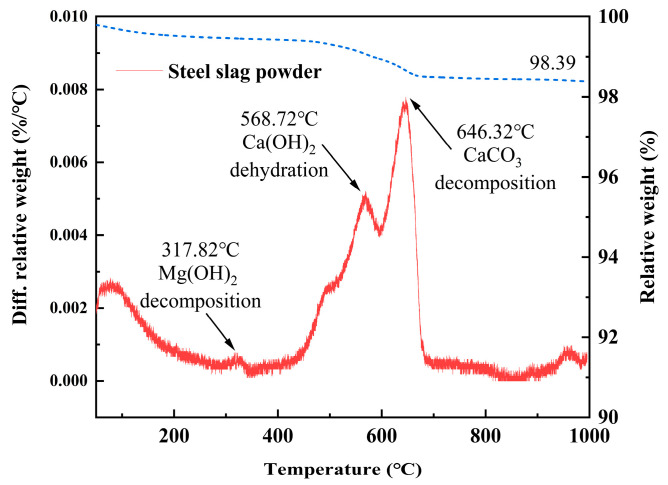
Thermal decomposition curve of steel slag powder.

The XRD pattern and the thermogravimetric (TG) and differential scanning calorimetry (DSC) curves of steel slag powder are shown in Figure 1 and Figure 2 respectively. In Figure 1, steel slag powder is mainly composed of C_2_S, C_2_F, SiO_2_ and CaO, with a small Ca(OH)_2_ content. In Figure 2, the first endothermic peak at approximately 317 °C corresponds to the evaporation of physically adsorbed free water and the decomposition of Mg(OH)_2_, indicating the loss of both physically adsorbed and chemically bound water in the steel slag powder. The second endothermic peak at around 568 °C is attributed to the dehydration of Ca(OH)_2_, reflecting the thermal decomposition of hydrated calcium phases in the slag. The prominent endothermic peak observed at 646 °C is related to the decarbonation of CaCO_3_ present in the steel slag, which involves the release of CO_2_ gas via thermal decomposition and represents the dominant weight-loss event in the TG curve. River sand (RS) comes from Wulian County, Rizhao City, Shandong Province, features a bulk density of 1506 kg/m^3^, a compaction density of 1658 kg/m^3^, an apparent density of 2429 kg/m^3^, a fineness modulus of 3.0, and a D_50_ of 942 μm. The steel fiber (F) is made of hook-shaped steel fiber microfiber with copper-plated ends (Shanghai Zhenqiang Fiber Co., Ltd., Shanghai, China), which has a tensile strength greater than 2000 MPa and a specification of 13 mm (length) × 0.22 mm (diameter). Water reducer (WRA) ad opts PCA^Ⓡ^-I superplasticizer of Jiangsu Subote New Materials Co., Ltd. (Nanjing, China), with solid content of 31% and water reduction rate of 31%. The water (W) is laboratory tap water.

### 2.2. Mixture Proportions and Specimen Preparation

Based on the theory of the closest packing of particles, the mixture ratio of UHPC designed by using the modified Andreasen & Andersen (MAA) model [28,29] is shown in Table 2. The mixture ratio is based on the reference group S0 [27], and steel slag powder with different fineness and dosage is used to replace cement to prepare UHPC mixed with steel slag powder. The subscripts of “S” respectively represent steel slag powder with different fineness (SSP 1, SSP 2 and SSP 3), and the following numbers represent the percentage content (20%, 30% and 40%) of steel slag powder. The particle-size distribution and UHPC accumulation curves for each raw material are shown as illustrated in Reference [27].

The preparation steps for steel slag powder UHPC are as follows: Firstly, cement, silica fume, fly ash microspheres, steel slag powder, and river sand were poured into a mixer and stirred for 2 min. Then the mixture of water and superplasticizer was added and stirred slowly for 5 min. The UHPC matrix was allowed to stand for 1–2 min. Finally, steel fiber was added and stirred for 4 min. The fresh UHPC was poured from the mixer into a mold, covered with plastic wrap, and placed in a natural curing condition (room temperature, ambient humidity) for 24 h. Then the mold was removed and placed under standard curing conditions: the temperature was 20 ± 2 °C, and the relative humidity was ≥95%.

### 2.3. Test Methods

#### 2.3.1. Workability Test

**Figure 3:**
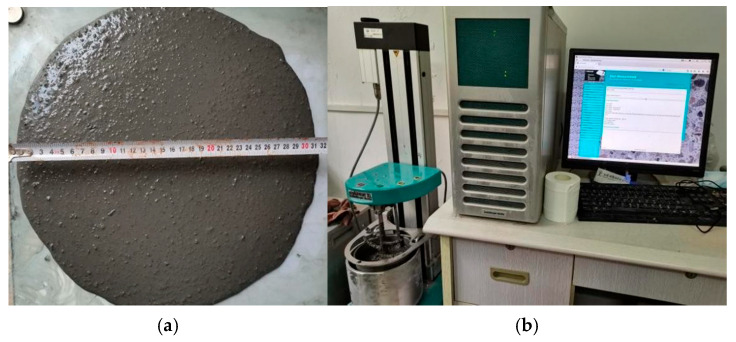
Workability test: (**a**) Fluidity test; (**b**) Rheological property test.

The workability of UHPC was assessed through fluidity and rheological property measurements (Figure 3). Fresh UHPC fluidity was quantified using slump flow tests in accordance with the Chinese standard JGJ/T 70-2009 [30]. Rheological characterization of the UHPC matrix was performed using a coaxial cylindrical rotational rheometer. Testing commenced 10 min after water addition, utilizing a 3 L sample volume. The rheological testing protocol is illustrated in Reference [27] and includes the following: Firstly, pre-shearing was performed for 1 min conditioning at 30 rpm to homogenize the mixture. Then the formal shear test was initiated after a 1 min rest period, employing eight shear rates (2, 6, 10, 14, 18, 22, 26, and 30 rpm). Shear rates were systematically increased from 2 to 30 rpm, then decreased inversely to evaluate thixotropic behavior. Each shear rate was maintained for 20 s, with 200 data points recorded per stage. The final 50 equilibrium data points at each shear rate were averaged to determine steady-state shear stress [31,32,33,34]. Finally, the Bingham model was applied to fit equilibrium shear stress values, with the fitting range restricted to 6–30 rpm to exclude potential plug-flow effects at lower shear rates (<6 rpm).

The relationship between torque (*T*, N∙mm) and rotational speed (*N*, rpm) during the downward section of the rheological test was fitted via linear regression [32], expressed in Equation (1):

(1) T = G + H·N

where *G* is the yield torque (yield-stress parameter, N∙mm), and *H* is the flow resistance (plastic-viscosity parameter, N·mm·min).

The relationship between yield stress (*τ*_0_, Pa) and plastic viscosity (*μ*, Pa·s) in the Bingham model [35] is expressed in Equation (2):

(2) $$\tau =\tau_{0}+\mu \cdot \dot{\gamma }$$

where *τ* is the shear stress (Pa), and $\dot{\gamma }$ is the shear rate (rpm).

The yield stress *τ*_0_ and plastic viscosity *μ* of the UHPC matrix can be calculated using the Reiner–Riwlin equation [36,37,38,39], as shown in Equations (3) and (4):

(3) $$\tau_{0}=\frac{\left(\frac{1}{{R_{1}}^{2}}-\frac{1}{{R_{2}}^{2}}\right)}{4\cdot \pi \cdot h\cdot ln(\frac{R_{2}}{R_{1}})}G$$

(4) $$\mu =\frac{\left(\frac{1}{{R_{1}}^{2}}-\frac{1}{{R_{2}}^{2}}\right)}{8\cdot \pi^{2}\cdot h}H$$

where *h* is the depth of the probe penetrated into the mix (fixed at 141 mm), *R*_1_ is the radius of the probe (73 mm), and *R*_2_ is the radius of the outer cylinder (82 mm).

#### 2.3.2. Mechanical Property Test

The compressive and tensile strength tests of the UHPC specimens were conducted in accordance with the T/CBMF 37-2018 standard [40], with three specimens per group. The cube specimens for the compressive strength test measured 100 mm × 100 mm × 100 mm, while the middle section of the dog-bone-shaped specimens for the tensile strength test measured 30 mm × 13 mm, as illustrated in Reference [27]. The mechanical properties of all specimens were tested after 28 d of standard curing.

#### 2.3.3. Microstructure Test

The sample was first submerged in anhydrous ethanol to terminate hydration, then ground into powder, and subsequently dried in a vacuum drying oven at 50 °C. X-ray diffraction (XRD) analysis was conducted using a SmartLab SE diffractometer produced by Rigaku Corporation, Tokyo, Japan, with CuK radiation employed for qualitative analysis of the matrix crystals. The scanning parameters were set as follows: a scanning speed of 2°/min, an angular accuracy of ±0.01°, and a scanning range of 5–80°.

The microstructure of hardened UHPC was analyzed using scanning electron microscopy (SEM) produced by Carl Zeiss AG, Jena, Germany. Samples with approximate dimensions of 25 mm^3^ was coated with gold and palladium. SEM imaging was conducted at magnifications ranging from 1000× to 5000× to resolve both bulk matrix features and interfacial transition zones.

## 3. Test Results and Analysis

### 3.1. Workability

#### 3.1.1. Fluidity

**Figure 4:**
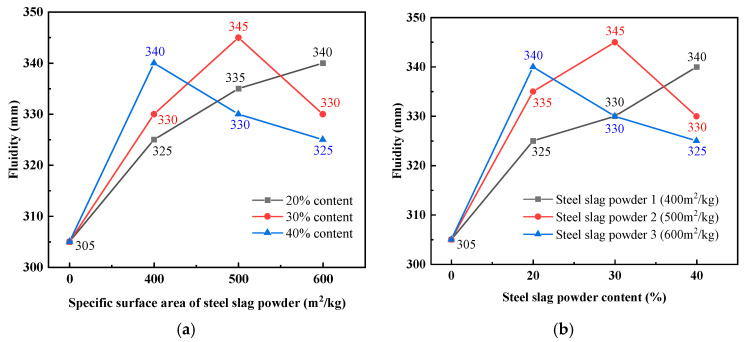
Effect of fineness and content of steel slag powder on fluidity of UHPC: (**a**) steel slag powder fineness; (**b**) content of steel slag powder.

Figure 4 shows the effect of the fineness and content of steel slag powder on the fluidity of UHPC. The fluidity of UHPC can be increased by adding steel slag powder. This is due to the low water demand of steel slag powder and the increased free water required for lubrication in the paste, which reduces the friction between particles and increases the fluidity of the UHPC matrix.

Figure 4a shows the effect of the fineness of steel slag powder on the fluidity of UHPC. When the steel slag powder content is 20%, the fluidity of UHPC increases with the increase in the fineness of steel slag powder. This is due to the pores in UHPC being better filled, thereby reducing the resistance that paste flow needs to overcome. When the content of steel slag powder is 30%, the fluidity of UHPC first increases and then decreases with the increase in the fineness of steel slag powder. This is because when the fineness of steel slag powder increases to a certain extent, the water demand increases and the cohesion between particles increases, which leads to a decrease in the fluidity of UHPC. Cohesion between particles implies yield stress, agglomeration, or inter-particle attraction. When the content of steel slag powder is 40%, the fluidity of UHPC decreases with the increase in fineness of steel slag powder. This is because when the content of steel slag powder is too high, the water demand increases sharply with the increase in fineness, and the cohesion between particles increases, which makes the fluidity of UHPC decrease.

Figure 4b shows the influence of the content of steel slag powder on the fluidity of UHPC. The fluidity of UHPC increases with the increase in the content of steel slag powder 1. With an increase in the content of steel slag powder 1, the water demand decreases, the free water content increases and the friction between particles decreases, resulting in an increase in UHPC fluidity. The fluidity of UHPC first increases and then decreases with the increase in the content of steel slag powder 2. This is because an appropriate amount of steel slag powder can fill the pores of UHPC and increase the fluidity of UHPC, but when the content is too high, irregular steel slag powder increases the shear force of the matrix, resulting in a decrease in the fluidity of UHPC. The fluidity of UHPC decreases gradually with the increase in the content of steel slag powder 3. This is mainly due to the large fineness of steel slag powder. With the increase in the content, the cohesion between particles also increases, resulting in a decrease in the fluidity of UHPC.

#### 3.1.2. Rheological Property

**Figure 5:**
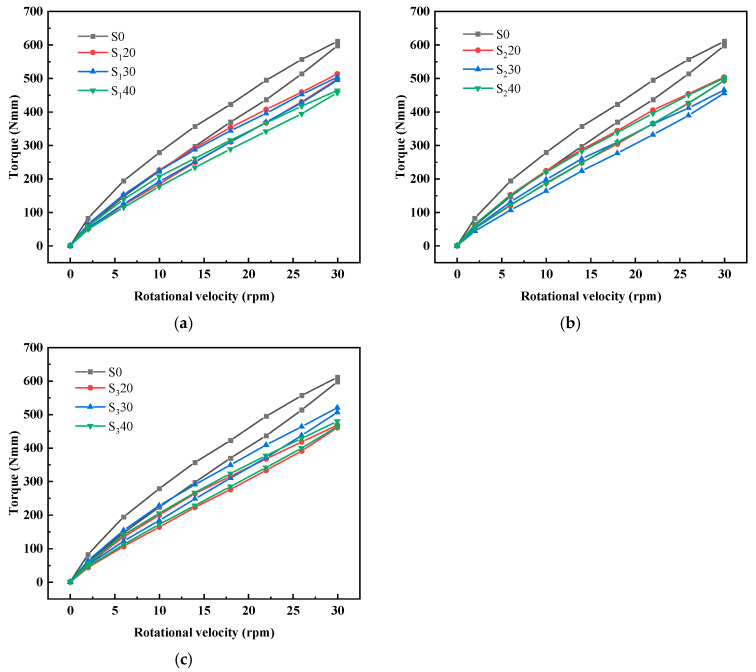
Torque–speed curve of UHPC mixed with steel slag powder: (**a**) steel slag powder 1; (**b**) steel slag powder 2; (**c**) steel slag powder 3.

Figure 5 illustrates the torque–speed relationship of UHPC incorporating steel slag powder, which characterizes the rheological response of fresh UHPC under shear loading. The Bingham model was applied to fit the linear region of the descending curve, and the derived rheological parameters (yield stress and plastic viscosity) are listed in Table 3. These parameters are critical indicators for evaluating the workability of fresh UHPC. Yield stress determines the minimum stress required to initiate flow, while plastic viscosity reflects the flow resistance during deformation. The data in Table 3 quantify the effects of steel slag powder content and specific surface area on the rheological properties of UHPC, offering fundamental insights for optimizing mix design and ensuring satisfactory construction performance of UHPC mixed with steel slag powder.

(1) Yield stress and plastic viscosity

**Figure 6:**
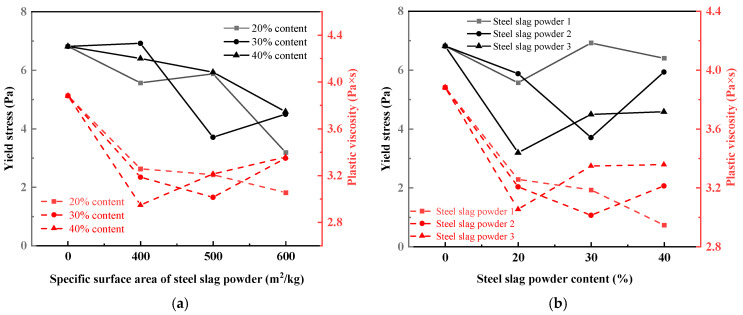
Effect of fineness and content of steel slag powder on rheological parameters of UHPC: (**a**) steel slag powder fineness; (**b**) content of steel slag powder.

Figure 6a shows the variation in yield stress and plastic viscosity of the UHPC matrix with the fineness of steel slag powder. When steel slag powder replaces 20% and 30% of the cement, the yield stress and plastic viscosity of UHPC matrix mixed with steel slag powder 3 and steel slag powder 2 are the smallest, and their fluidity is the highest respectively. When steel slag powder replaces 40% of the cement, the ability of the UHPC matrix to maintain its original state gradually weakens with the increase in steel slag powder fineness. However, when the flow of the UHPC matrix occurs, the viscosity between the layers gradually increases, and the fluidity gradually decreases, which is consistent with the previous rule. The yield stress and plastic viscosity of the UHPC matrix mixed with steel slag powder are not the same as increasing and decreasing. This is because the yield stress reflects the minimum force required for the UHPC matrix to flow, which is related to the consistency. In comparison, the plastic viscosity reflects the internal friction of the relative sliding between the layers after the flow of the UHPC matrix, which is the embodiment of mixture viscosity.

Figure 6b shows the variation in yield stress and plastic viscosity of the UHPC matrix with the content of steel slag powder. The ability of the UHPC matrix to maintain its original state was enhanced with the increase in steel slag powder 1. However, when the flow of the UHPC matrix occurs, the viscosity between the layers decreases, so the fluidity increases. With the increase in the content of steel slag powder 2, the yield stress and plastic viscosity of the UHPC matrix decrease first and then increase, which is consistent with the fluidity rule. The yield stress and plastic viscosity of the UHPC matrix increase gradually with the increase in steel slag powder 3 content, and the fluidity of the UHPC matrix decreases gradually.

(2) Thixotropy

Thixotropic behavior describes a material’s time-dependent response under shear forces, manifested by decreasing apparent viscosity and concurrently increasing shear strain rate as applied stress escalates. This rheological property serves as an indicator for evaluating the structural recovery capacity of UHPC matrices following deformation. Contemporary characterization techniques primarily employ two approaches: hysteresis-loop analysis and shear-stress attenuation methods. The hysteresis-loop methodology involves generating a closed curve through cyclic shear rate variations, formed by contrasting torque–speed relationships during the ascending and descending rotational phases [27]. The magnitude of this enclosed region quantitatively represents the material’s thixotropic characteristics [41,42]. Experimental data presented in Table 3 demonstrate the hysteretic-loop measurements for the UHPC matrix mixed with steel slag powder, where rotational speeds between 6 and 30 rpm were systematically applied to determine loop areas.

The thixotropic behavior of the UHPC matrix was evaluated via a shear-stress attenuation method, quantified through a dimensionless thixotropic index. Under a constant shear rate, torque within the UHPC system exhibited an initial surge to maximum values followed by a gradual decay toward an equilibrium state [27]. The thixotropic index *I_thix_* is defined as the ratio of initial to equilibrium stress parameters:

(5) $$I_{thix}=\frac{\tau_{i}}{\tau_{e}}=\frac{\Gamma_{i}}{\Gamma_{e}}$$

where *τ_i_* and *τ_e_* represent the initial yield stress and the equilibrium stress, respectively. Correspondingly, *Γ_i_* denotes the initial maximum torque during stress application, while *Γ_e_* signifies the equilibrium torque of stress attenuation derived from averaging the final 50 measurements preceding stabilization.

**Figure 7:**
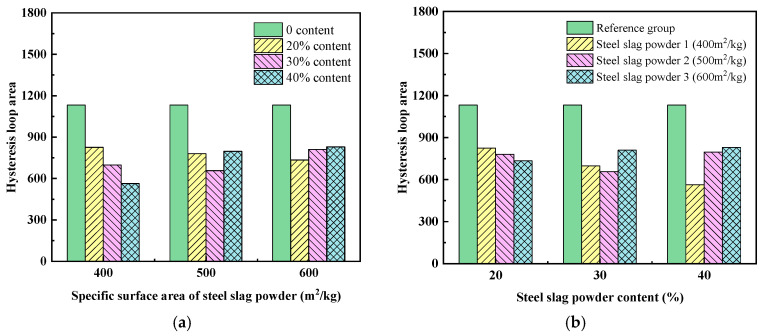
Effect of steel slag powder fineness and content on the hysteresis loop area of UHPC: (**a**) fineness of steel slag powder; (**b**) content of steel slag powder.

**Figure 8:**
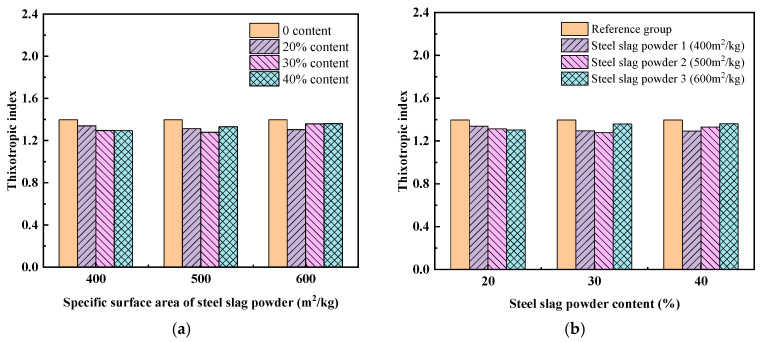
Effect of steel slag powder fineness and content on thixotropic index of UHPC: (**a**) fineness of steel slag powder; (**b**) content of steel slag powder.

Figure 7a and Figure 8a show the influence of the fineness of steel slag powder on the hysteresis loop area and the thixotropic index of UHPC, and the rules of the two figures are basically consistent. When the content of steel slag powder is 20%, the hysteretic loop area and thixotropic index of the UHPC matrix gradually decrease with the increase in the fineness of steel slag powder, indicating that the thixotropy recovery ability of the UHPC matrix decreases. When the content of steel slag powder is 30%, the hysteresis loop area and thixotropic index decrease first and then increase with the increase in the fineness of steel slag powder, indicating that the thixotropy recovery ability of the UHPC matrix decreases first and then increases. When the content of steel slag powder is 40%, the hysteresis loop area and thixotropic index of the UHPC matrix gradually increase with the increase in the fineness of steel slag powder, indicating that the thixotropy recovery ability of the UHPC matrix is improved.

Figure 7b and Figure 8b show the influence of steel slag powder content on hysteresis loop area and thixotropy index of UHPC. The hysteresis loop area and thixotropic index of UHPC decrease gradually with the increase in the content of steel slag powder 1, indicating that the thixotropy recovery ability of UHPC decreases. The hysteresis loop area and thixotropic index decrease first and then increase with the increase in the content of steel slag powder 2, indicating that the thixotropy recovery ability also decreases first and then increases. The hysteresis loop area and thixotropic index of the UHPC matrix increase gradually with the increase in the content of steel slag powder 3, indicating that the thixotropy recovery ability of the UHPC matrix is improved.

### 3.2. Mechanical Property

#### 3.2.1. Compressive Strength

**Figure 9:**
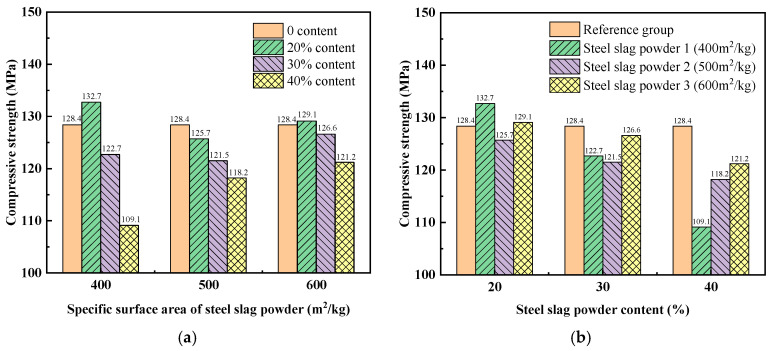
Effect of fineness and content of steel slag powder on compressive strength of UHPC: (**a**) fineness of steel slag powder; (**b**) content of steel slag powder.

The influence of the fineness of steel slag powder on the compressive strength of UHPC is shown in Figure 9a. The compressive strength of UHPC mixed with only 20% steel slag powder 1 and steel slag powder 3 is 3.35% and 0.55% higher than that of the reference group, respectively. When adding 20% and 30% steel slag powder, the compressive strength of UHPC decreases first and then increases with the increase in the fineness of steel slag powder, but the change is not large. When 40% steel slag powder is added, the compressive strength of UHPC increases gradually with the increase in the fineness of steel slag powder. This is because the finer steel slag powder has higher activity and can fully leverage the micro-aggregate effect and pozzolanic effect, thereby filling the tiny pores of UHPC to increase its compactness, thus increasing the compressive strength.

The influence of steel slag powder content on the compressive strength of UHPC is shown in Figure 9b. For UHPC mixed with any fineness of steel slag powder, the compressive strength of UHPC decreases with the increase in the mixing content. This is because the increase in the content of steel slag powder leads to a decrease in the content of cement, and the generated hydration products also decrease, resulting in a decrease in the compressive strength of UHPC. Among them, the compressive strength of UHPC mixed with steel slag powder 1 decreased the most, because it had coarse particles and low activity, which also had the greatest negative impact on UHPC strength. The compressive strength of UHPC mixed with 40% steel slag powder 1 was only 109.1 MPa, which was 15.03% lower than that of the reference group.

#### 3.2.2. Axial Tensile Strength

**Figure 10:**
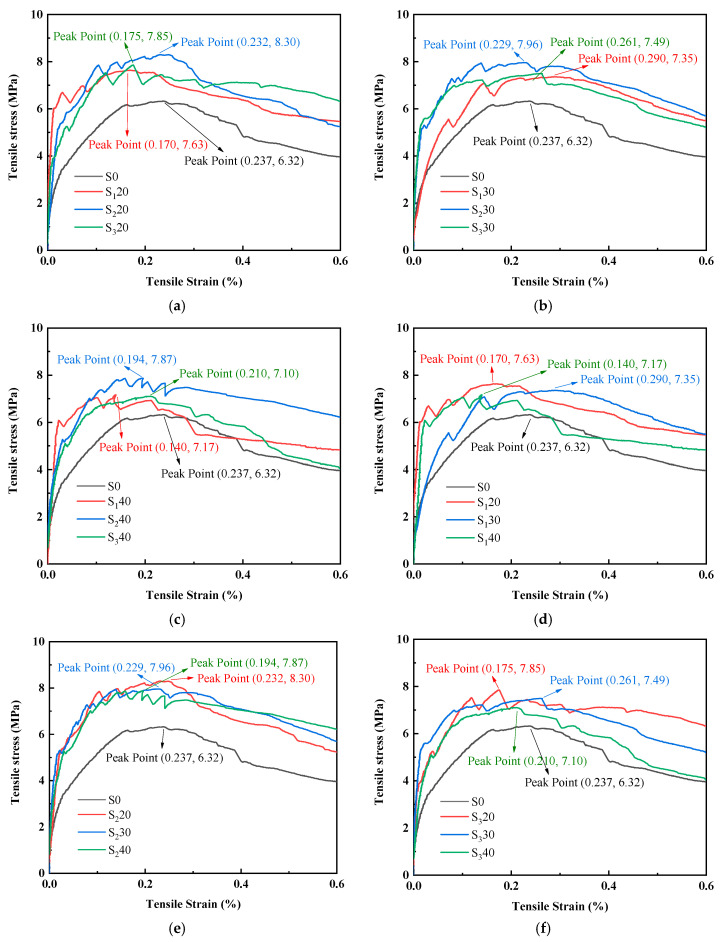
Tensile stress–strain curve of UHPC mixed with steel slag powder: (**a**) fineness of steel slag powder (20% content); (**b**) fineness of steel slag powder (30% content); (**c**) fineness of steel slag powder (40% content); (**d**) content of steel slag powder 1; (**e**) content of steel slag powder 2; (**f**) content of steel slag powder 3.

Figure 10 shows the tensile stress-strain curve of UHPC with different fineness and content of steel slag powder. The tensile strength of UHPC mixed with steel slag powder is higher than that of the reference group, indicating that mixing steel slag powder can improve the tensile properties of UHPC. The tensile stress–strain curve of UHPC has typical strain hardening characteristics, which can be roughly divided into three stages. The first stage is a linear-elastic ascending section, with UHPC cracking as the end sign, and the initial cracking strength mainly reflects the strength of the UHPC matrix. The second stage is the rising stage of strain hardening, with the peak tensile strength of UHPC as the end sign. The third section is the softening and descending section.

Figure 10a–c are UHPC stress–strain curves of steel slag powder with different fineness at the same content. When the content of steel slag powder is increased from 20% to 40%, the tensile strength of UHPC mixed with steel slag powder 1 and steel slag powder 3 is similar. The tensile strength of UHPC mixed with steel slag powder 1 was 20.73%, 16.30% and 13.45% higher than that of the reference group, respectively. The tensile strength of UHPC mixed with steel slag powder 2 was the highest, which was 31.33%, 25.95% and 24.53% higher than that of the reference group, respectively.

Figure 10d–f are UHPC stress–strain curves with the same fineness and different contents of steel slag powder. The tensile strength of UHPC decreases with the increase in steel slag powder of any fineness.

### 3.3. Microstructure Analysis

#### 3.3.1. Hydration Product Analysis

**Figure 11:**
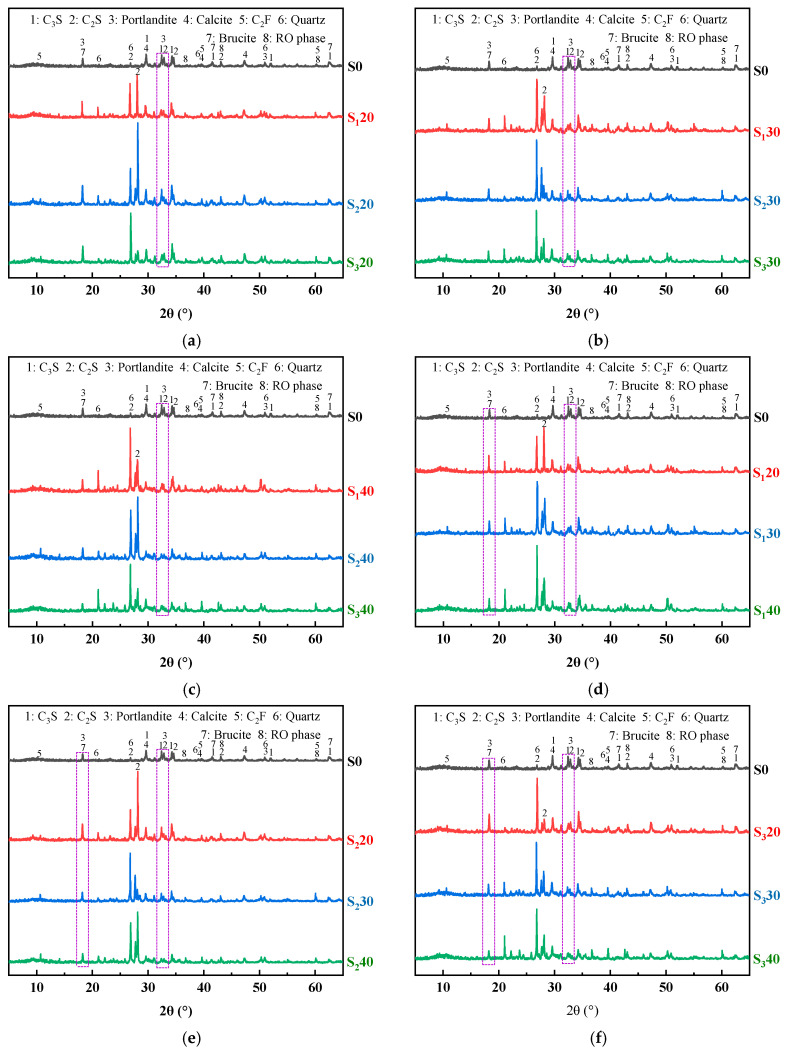
XRD pattern of UHPC mixed with steel slag powder: (**a**) fineness of steel slag powder (20% content); (**b**) fineness of steel slag powder (30% content); (**c**) fineness of steel slag powder (40% content); (**d**) content of steel slag powder 1; (**e**) content of steel slag powder 2; (**f**) content of steel slag powder 3.

Figure 11 shows the XRD pattern of UHPC mixed with steel slag powder. The shapes of each experimental group are similar, and the main changes are the diffraction peaks of Ca(OH)_2_, Mg(OH)_2_, C_3_S, C_2_S, SiO_2_ and RO phases. The C_3_S and C_2_S diffraction peaks of UHPC mixed with steel slag powder are still obvious, indicating that there is a certain amount of unhydrated cement particles, which provides a theoretical basis for preparing UHPC with steel slag powder instead of cement. Because the steel slag powder also contains Ca(OH)_2_, the content of Ca(OH)_2_ in some experimental groups is higher than that in the reference group, but due to its larger crystal size, which adversely affects the mechanical properties of UHPC.

Figure 11a–c are XRD patterns of steel slag powder UHPC with different fineness at the same content. When the content of steel slag powder is 20% and 30% respectively, the diffraction peaks of C_3_S and C_2_S in the experimental groups mixed with steel slag powder 1 and steel slag powder 3 are lower. This indicates that C_3_S and C_2_S fully react at this time to generate more hydration products, making their compressive strength higher. The diffraction peaks of C_3_S and C_2_S in the test group mixed with steel slag powder 2 are higher, so the compressive strength is slightly worse. When the content of steel slag powder is 40%, C_3_S and C_2_S in the experimental group mixed with steel slag powder 1 are not completely hydrated, so the compressive strength decreases significantly.

Figure 11d–f are XRD patterns of UHPC with the same fineness and different contents of steel slag powder. The diffraction peaks of Ca(OH)_2_ and Mg(OH)_2_ of UHPC gradually decrease with the increase in steel slag powder content when steel slag powder with any fineness is added. These results indicate that the Ca(OH)_2_, Mg(OH)_2_ and ettringite crystals generated by UHPC gradually decrease, which is not conducive to the development of matrix strength, so the compressive strength gradually decreases.

#### 3.3.2. Micromorphology Analysis

**Figure 12:**
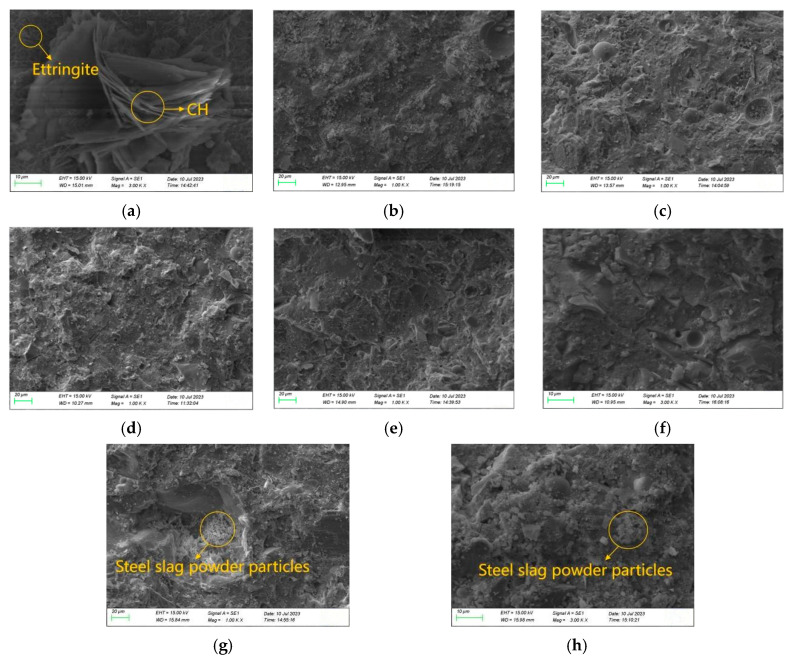
Micromorphology of UHPC mixed with steel slag powder: (**a**) S0-28 d; (**b**) S_1_20-28 d; (**c**) S_2_20-28 d; (**d**) S_3_20-28 d; (**e**) S_1_30-28 d-1; (**f**) S_1_30-28 d-2; (**g**) S_1_40-28 d-1; (**h**) S_1_40-28 d-2.

Figure 12 shows the micromorphology of a UHPC matrix mixed with steel slag powder after standard curing for 28 days. As UHPC has an extremely low water–cement ratio, there are numerous unhydrated cement particles in the reference group [27]. Figure 12a also shows plate-like Ca(OH)_2_ and needle-like ettringite in the reference group. Figure 12b–d show the influence of steel slag powder with different fineness on the morphology of UHPC hydration products when the content is 20%. When steel slag powder of any fineness is added, the microstructure of UHPC is relatively dense, and a large number of C-S-H gels are f generated, but there are still unhydrated binder particles on the surface. It shows that the water–binder ratio is still low at this time, which further proves that it is reasonable and feasible to use a certain amount of steel slag powder instead of cement to prepare UHPC. Among them, the UHPC mixed with steel slag powder 1 has the fewest defects and the highest 28 d compressive strength.

Figure 12b,e–h compared the product morphology of UHPC with different content of steel slag powder 1. When the content of steel slag powder 1 is 20%, the microstructure of UHPC is the densest, so the matrix strength is the highest. When the content of steel slag powder 1 is 30%, the surface defects and pores of UHPC increase, which is not conducive to the development of matrix strength. When the content of steel slag powder is 40%, a large number of unhydrated steel slag powder particles appear on the surface of UHPC, and the hydration products are insufficient, resulting in increased porosity. The density of the UHPC microstructure is further affected, so its strength is the lowest.

## 4. Conclusions

In this study, steel slag powder with different fineness and content was used to replace cement in UHPC as a mineral admixture. The effect of this steel slag powder on the workability, mechanics, and microstructure of UHPC was investigated, and the following conclusions were inferred:

(1) The results of rheological properties of UHPC show that the addition of steel slag powder can significantly reduce the yield stress and plastic viscosity of UHPC, thereby increasing its fluidity, but decreasing its thixotropy.
(2) The tensile properties of UHPC mixed with steel slag powder were better than those of the reference group. For instance, the tensile strengths of UHPC with 20%, 30% and 40% content of steel slag powder 1 (with a specific surface area of 400 m^2^/kg) were 20.73%, 16.30% and 13.45% higher than those of the reference group, respectively.
(3) The compressive strength of the experimental group mixed with steel slag powder 3 (with a specific surface area of 600 m^2^/kg) was higher as a whole, because the finer steel slag powder has higher activity, and can give full play to the micro-aggregate effect and pozzolanic effect to fill the tiny pores of UHPC. Then the compactness of the UHPC matrix was increased, and the compressive strength was increased. When 20% content of the cement was replaced by steel slag powder 1, the compressive strength of UHPC was 3.35% higher than that of the reference group. This was because C_3_S and C_2_S underwent sufficient hydration reactions to generate more hydration products at this time.
(4) When the steel slag powder of any fineness was added, the mechanical properties of UHPC gradually decreased with the increase in the content. This is because an increase in steel slag powder content leads to a decrease in cement content, and the hydration products generated also decrease, resulting in a decrease in the microstructure density and strength of UHPC.
(5) It is recommended to use steel slag powder 1 to replace 20% cement in UHPC. At this time, the workability and mechanical properties of UHPC are better than those of the reference group, and the fineness of the steel slag powder is lower, making it more economical, low-carbon and environmentally friendly.

## Figures and Tables

**Table 1:** Main chemical compositions of SSP (%).

Materials	CaO	SiO_2_	Al_2_O_3_	MgO	SO_3_	Fe_2_O_3_	K_2_O	Na_2_O	P_2_O_5_	TiO_2_
SSP	5.15	61.48	12.51	3.23	-	6.90	5.04	3.48	0.49	0.76

**Table 2:** Mixture proportion of UHPC mixed with steel slag powder (kg/m^3^).

Groups	CEM	SF	FAM	SSP 1	SSP 2	SSP 3	RS	W	WRA	F
S0	801	128	139	0	0	0	1067	192	21.4	156
S_1_20	640.8	128	139	160.2	0	0	1067	192	21.4	156
S_1_30	560.7	128	139	240.3	0	0	1067	192	21.4	156
S_1_40	480.6	128	139	320.4	0	0	1067	192	21.4	156
S_2_20	640.8	128	139	0	160.2	0	1067	192	21.4	156
S_2_30	560.7	128	139	0	240.3	0	1067	192	21.4	156
S_2_40	480.6	128	139	0	320.4	0	1067	192	21.4	156
S_3_20	640.8	128	139	0	0	160.2	1067	192	21.4	156
S_3_30	560.7	128	139	0	0	240.3	1067	192	21.4	156
S_3_40	480.6	128	139	0	0	320.4	1067	192	21.4	156

**Table 3:** Rheological performance parameters of UHPC mixed with steel slag powder.

Groups	G (Nmm)	H (Nmm·min)	Yield Stress (Pa)	Plastic Viscosity (Pa·s)	Relevance	Hysteresis Loop Area (Physics)	Thixotropic Index
S0	36.08	18.50	6.82	3.88	0.9992	1131.60	1.396
S_1_20	29.47	15.52	5.57	3.26	0.9996	825.12	1.339
S_1_30	36.62	15.18	6.92	3.18	0.9994	697.28	1.296
S_1_40	33.88	14.04	6.40	2.95	0.9992	563.76	1.294
S_2_20	31.11	15.28	5.88	3.21	0.9993	779.76	1.313
S_2_30	19.62	14.36	3.71	3.01	0.9991	656.88	1.278
S_2_40	31.41	15.31	5.94	3.21	0.9996	796.08	1.331
S_3_20	16.89	14.55	3.19	3.05	0.9987	733.68	1.304
S_3_30	23.77	15.96	4.49	3.35	0.9996	810.00	1.359
S_3_40	24.25	14.52	4.58	3.05	0.9996	828.88	1.361

## Data Availability

The original contributions presented in this study are included in the article. Further inquiries can be directed to the corresponding author.

## References

[1] Gao W., Zhou W., Lyu X., Liu X., Su H., Li C., Wang H. (2023). Comprehensive utilization of steel slag: A review. Powder Technol..

[2] Guo J., Bao Y., Wang M. (2018). Steel slag in China: Treatment, recycling, and management. Waste Manag..

[3] Shi C. (2002). Characteristics and cementitious properties of ladle slag fines from steel production. Cem. Concr. Res..

[4] Bache H.H. Densified cement/ultra fine particle based materials. Proceedings of the Second International Conference on Superplasticizers in Concrete.

[5] Larrard F.D., Sedran T. (1994). Optimization of ultra-high-performance concrete by the use of a packing model. Cem. Concr. Res..

[6] Yu R., Spiesz P., Brouwers H.J.H. (2014). Mix design and properties assessment of ultra-high performance fibre reinforced concrete (UHPFRC). Cem. Concr. Res..

[7] Brühwiler E. (2020). UHPFRC technology to enhance the performance of existing concrete bridges. Struct. Infrastruct. Eng..

[8] Zhang X., Zhao S., Liu Z., Wang F. (2019). Utilization of steel slag in ultra-high performance concrete with enhanced eco-friendliness. Constr. Build. Mater..

[9] Yüksel İ. (2017). A review of steel slag usage in construction industry for sustainable development. Environ. Dev. Sustain..

[10] Han F., Zhang Z. (2018). Properties of 5-year-old concrete containing steel slag powder. Powder Technol..

[11] Pan Z., Zhou J., Jiang X., Xu Y., Jin R., Ma J., Zhuang Y., Diao Z., Zhang S., Si Q. (2019). Investigating the effects of steel slag powder on the properties of self-compacting concrete with recycled aggregates. Constr. Build. Mater..

[12] Zang J., Li W., Shen X. (2019). The influence of steel slag with variable particle size distribution on the workability and mechanical properties of concrete. Ceram.-Silikáty.

[13] Ming Y., Chen P., Wang Y., Li L., Chen X., Sun P. (2020). Experimental research of concrete with steel slag powder and Zeolite powder. J. Renew. Mater..

[14] Han X., Feng J., Shao Y., Hong R. (2020). Influence of a steel slag powder-ground fly ash composite supplementary cementitious material on the chloride and sulphate resistance of mass concrete. Powder Technol..

[15] Fang M., Fang G., Xia Y., Wang H. (2020). Study on Compressive Strength of Concrete Mixed by Steel Slag Powder and Fly Ash. IOP Conf. Ser. Earth Environ. Sci..

[16] Zhuo K., Liu G., Lan X., Zheng D., Wu S., Wu P., Lin J. (2022). Fracture behavior of steel slag powder-cement-based concrete with different steel-slag-powder replacement ratios. Materials.

[17] Martins A.C.P., de Carvalho J.M.F., do Nascimento Duarte M., de Lima G.E.S., Pedroti L.G., Peixoto R.A.F. (2022). Influence of a LAS-based modifying admixture on cement-based composites containing steel slag powder. J. Build. Eng..

[18] Zhan P., Zhang X., He Z., Shi J., Gencel O., Yen N.T.H., Wang G. (2022). Strength, microstructure and nanomechanical properties of recycled aggregate concrete containing waste glass powder and steel slag powder. J. Clean. Prod..

[19] Tian E., Liu Y., Cheng X., Zeng W. (2022). Characteristics of pavement cement concrete incorporating steel slag powder. Adv. Mater. Sci. Eng..

[20] Wang P., Xie M., Liu L. (2022). Study on Early Shrinkage and Mechanical Properties of Concrete with Various Cementitious Materials. Buildings.

[21] Zhao J., Li Z., Wang D., Yan P., Luo L., Zhang H., Zhang H., Gu X. (2023). Hydration superposition effect and mechanism of steel slag powder and granulated blast furnace slag powder. Constr. Build. Mater..

[22] Liu J., Guo R. (2018). Applications of steel slag powder and steel slag Aggregate in Ultra-High Performance Concrete. Adv. Civ. Eng..

[23] Peng Y., Chen K., Hu S. (2011). Durability and microstructure of ultra-high performance concrete having high volume of steel slag powder and ultra-fine fly ash. Adv. Mater. Res..

[24] Fan D., Yu R., Shui Z., Liu K., Feng Y., Wang S., Li K., Tan J., He Y. (2021). A new development of eco-friendly Ultra-High performance concrete (UHPC): Towards efficient steel slag application and multi-objective optimization. Constr. Build. Mater..

[25] Li S., Cheng S., Mo L., Deng M. (2020). Effects of steel slag powder and expansive agent on the properties of ultra-high performance concrete (UHPC): Based on a case study. Materials.

[26] (2008). Testing method for specific surface of cement — Blaine method.

[27] Liu L., Chen H., He Z., Wang P., Cui J., Cai X., Sun Y. (2024). Effect and mechanism of compound expansive agent on rheological, mechanical, and shrinkage properties of UHPC. Constr. Build. Mater..

[28] Wang J., Yu R., Ji D., Tang L., Yang S., Fan D., Shui Z., Leng Y., Liu K. (2022). Effect of distribution modulus (q) on the properties and microstructure development of a sustainable Ultra-High Performance Concrete (UHPC). Cem. Concr. Compos..

[29] Brouwers H.J.B., Radix H.J. (2005). Self-Compacting Concrete: Theoretical and experimental study. Cem. Concr. Res..

[30] (2009). Standard for Test Method of Performance on Building Mortar.

[31] Zhang C., Zhang X., Hou J., Wang J., Duan G. (2022). Rheology and early microstructure evolution of fresh ultra-high performance concrete with polycarboxylate superplasticizer. Case Stud. Constr. Mater..

[32] Liu J., An M., Wang Y., Han S., Yu Z. (2022). Research on the relation between slump flow and yield stress of Ultra-high performance concrete mixtures. Materials.

[33] Khayat K.H., Meng W., Vallurupalli K., Teng L. (2019). Rheological properties of ultra-high-performance concrete—An overview. Cem. Concr. Res..

[34] Zuo S., Yuan Q., Huang T., Wang Z., Zhang K., Liu J. (2023). Rheology and air entrainment of fresh Portland cement mortars in simulated low air pressure environments. Cem. Concr. Compos..

[35] Li K., Leng Y., Xu L., Zhang J., Liu K., Fan D., Yu R. (2022). Rheological characteristics of Ultra-High performance concrete (UHPC) incorporating bentonite. Constr. Build. Mater..

[36] Feys D., Wallevik J.E., Yahia A., Khayat K.H., Wallevik O.H. (2013). Extension of the Reiner-Riwlin equation to determine modified Bingham parameters measured in coaxial cylinders rheometers. Mater. Struct..

[37] Weng Y., Li M., Tan M., Qian S. (2018). Design 3D printing cementitious materials via Fuller Thompson theory and Marson-Percy model. Constr. Build. Mater..

[38] Nerella V.N., Näther M., Iqbal A., Butler M., Mechtcherine V. (2019). Inline quantification of extrudability of cementitious materials for digital construction. Cem. Concr. Compos..

[39] Zhu H., Zhang D., Li V.C. (2022). Centrifugally sprayed Engineered Cementitious Composites: Rheology, mechanics, and structural retrofit for concrete pipes. Cem. Concr. Compos..

[40] (2018). Fundamental Characteristics and Test Methods of Ultra-High Performance Concrete.

[41] Cui J., He Z., Zhang G., Cai X., Hu L. (2022). Rheology, mechanical properties and pore structure of sprayed ultra-high performance concrete (SUHPC) with viscosity-enhancing agent. Constr. Build. Mater..

[42] Cui J., He Z., Zhang G., Cai X. (2022). Rheological properties of sprayable ultra-high performance concrete with different viscosity-enhancing agents. Constr. Build. Mater..

